# 2-BFI Provides Neuroprotection Against Inflammation and Necroptosis in a Rat Model of Traumatic Brain Injury

**DOI:** 10.3389/fnins.2019.00674

**Published:** 2019-06-26

**Authors:** Haibo Ni, Qin Rui, Xiaolong Lin, Di Li, Huixiang Liu, Gang Chen

**Affiliations:** ^1^Department of Neurosurgery, Zhangjiagang First People’s Hospital, Suzhou, China; ^2^Department of Laboratory, Zhangjiagang First People’s Hospital, Suzhou, China; ^3^Department of Orthopedics, Zhangjiagang First People’s Hospital, Suzhou, China; ^4^Department of Translational Medicine Center, Zhangjiagang First People’s Hospital, Suzhou, China; ^5^Department of Neurosurgery and Brain and Nerve Research Laboratory, The First Affiliated Hospital of Soochow University, Suzhou, China

**Keywords:** 2-BFI, inflammation, necroptosis, neuroprotection, traumatic brain injury

## Abstract

Inflammation and programmed necrosis (necroptosis) are the two hallmark pathological changes after traumatic brain injury (TBI) that contribute to aggravated brain damage. 2-(2-Benzofuranyl)-2-imidazoline (2-BFI) has been shown to exert both anti-inflammatory and programmed cell death effects. Therefore, the aim of the present study was to evaluate the potential beneficial effects of 2-BFI in a rat model of TBI induced by a weight-drop device. 2-BFI or vehicle was given via intraperitoneal injection starting at 30 min post trauma and then twice daily for three consecutive days. Following a neurofunctional test at 72 h after injury, histological, molecular, and immunohistochemistry analyses were performed on the pericontusional areas of the brain. 2-BFI treatment significantly attenuated neurological deficits, brain edema and blood-brain barrier permeability after TBI. Also, treatment with 2-BFI significantly reduced microglial activation, neutrophil infiltration, and proinflammatory cytokine interleukin (IL)-1β secretion, which is related to nucleotide binding and oligomerization domain-like receptor family pyrin domain-containing 3 (NLRP3) inflammasome activation after TBI. In addition, 2-BFI treatment markedly reduced cortical tissue loss as well as repressed TBI-induced increases in necroptosis and necroptosis-associated proteins, including receptor-interacting protein (RIP1), RIP3, and mixed linkage kinase domain-like (MLKL) in the pericontusional brain tissue. Taken together, these findings indicate that 2-BFI may be an effective neuroprotectant after brain trauma and warrants further study.

## Introduction

Traumatic brain injury (TBI) is a major cause of mortality and morbidity among individuals younger than 45 years worldwide (Corrigan et al., 2010). The TBI mechanism is a complicated pathological process, with an initial mechanical injury that triggers biochemical and cellular changes that contribute to ongoing neurodegeneration and neurologic impairments. This delayed damage is known as secondary injury, which involves glutamate excitotoxicity, oxidative stress, inflammatory reaction and activation of cell death programs (Nizamutdinov and Shapiro, 2017). The combined actions of these mechanisms ultimately determine the prognosis and outcome of the trauma. Thus, a novel, safe, and effective drugs that act on multiple targets are urgently required for patients with TBI (Kumar and Loane, 2012; Loane et al., 2015).

Inflammation is extensively identified as a major contributor to the pathophysiology of secondary TBI damage (Kumar and Loane, 2012). The neuroinflammatory cascade activated in response to TBI is mediated by the release of proinflammatory cytokines, such as interleukin-1β (IL-1β) IL-6, and IL-18 (Lozano et al., 2015). Upregulation of these cytokines increases the permeability of the blood-brain barrier (BBB) and causes microglial activation, which results in inflammatory amplification that further contributes to secondary neurological injury (Kumar and Loane, 2012; Hernandez-Ontiveros et al., 2013; Lozano et al., 2015). Although not extensively studied, there is growing evidence that posttraumatic activation of the nucleotide binding and oligomerization domain-like receptor family pyrin domain-containing 3 (NLRP3) inflammasome axis can control the maturation and release of proinflammatory cytokines and is correlated with TBI severity (Liu et al., 2013; Irrera et al., 2017; Mortezaee et al., 2018).

Necrotic cell death is also a hallmark feature in the pathological processes of TBI, yet it lacks a specific target for therapeutic intervention because necrotic cell death has long been described as an accidental and unregulated cellular event (Werner and Engelhard, 2007). Recently, a type of programmed necrosis, named necroptosis, has been defined, and its associated signaling pathways have been gradually revealed (Vandenabeele et al., 2010). Molecularly, receptor-interacting protein 1 (RIP1) and 3 (RIP3) and their substrate, mixed lineage kinase domain-like (MLKL), are essential regulators of necroptosis (Li et al., 2012a; Sun et al., 2012). Both chemical inhibition of RIP1 and deletion of RIP3 in rodent models of TBI have been shown to alleviate brain tissue damage as well as contribute to an increased resistance to inflammatory responses, thus providing a novel therapeutic strategy for TBI (You et al., 2008; Liu et al., 2016, 2018).

2-(2-Benzofuranyl)-2-imidazoline (2-BFI), a selective imidazoline I_2_ receptor (I_2_R) agonist, has been viewed as an effective analgesic for persistent and chronic painful conditions (Li et al., 2011; Li, 2017). In recent years, 2-BFI has also been shown to exhibit promising neuroprotective properties in several central nervous system disease models, such as stroke (Han et al., 2013), focal cerebral ischemia (Han et al., 2010, 2012; Zhang et al., 2018), autoimmune encephalomyelitis (Wang et al., 2011; Li et al., 2012b; Zhu et al., 2015), and neurodegenerative diseases (Tian et al., 2017b). A number of *in vitro* and *in vivo* studies have demonstrated that these diverse effects of 2-BFI are due to its pleiotropic nature, especially on antiinflammatory and programmed cell death activities (Li, 2017; Tian et al., 2017b; Siemian et al., 2018). Given that the multipathological processes involved in secondary brain injury after TBI are targeted by 2-BFI, we therefore tested the hypothesis that 2-BFI also protects against TBI by affecting inflammatory and programmed cell death pathways in a rat model.

## Materials and Methods

### Animals

Male adult Sprague-Dawley rats (280–300 g) were purchased from the Animal Center of Chinese Academy of Sciences (Shanghai, China). All procedures were conducted in strict accordance with the ARRIVE Guidelines (Animal Research: Reporting of *In Vivo* Experiments) and approved by the Animal Care and Use Committee of Soochow University. The rats were housed in the animal facility under standard conditions under a 12-h light/dark cycle with food and water *ad libitum*. All efforts were made to minimize the number of animals used and the stress and pain of the animals.

### TBI Induction

A focal cortical contusion injury model induced by the weight-drop method was used to cause brain trauma, as described in our previous study (Ni et al., 2018). Briefly, general anesthesia was induced with 4% chloral hydrate (400 mg/kg, intraperitoneal injection). The rats were then placed in a stereotactic frame (Kopf Instruments, United States), and a 5-mm craniotomy was performed on the right side midway between the bregma and the lambda, with the medial edge of the craniotomy 1.0 mm lateral to the midline, leaving the dura intact. Brain trauma was induced using a weight-drop device comprising a metal rod (with a tip 4 mm in diameter and 5 mm in length) weighing 40 g that fell from a height of 25 cm directly onto the exposed skull. After injury, the incision was closed with wound clips. Sham-operated rats underwent identical surgical procedures except without the impaction. Body temperature was maintained at 37°C using a small-animal temperature controller throughout all procedures. All animals were allowed to completely recover from anesthesia in a warm chamber before they were returned to their home cages.

### Tissue Collection

All animals were deeply anesthetized with sodium pentobarbital (100 mg/kg), and the brains were collected on ice. For the isolation of proteins and mRNAs, samples of the pericontusional cortex of the brain located less than 3 mm from the margin of the contusion site in TBI rats and tissue samples from the same location in sham-operated rats were dissected and rapidly stored at −80°C. For coronal sections, the tissue was postfixed in 4% paraformaldehyde overnight at 4°C and cryoprotected in 20% and then 30% sucrose in phosphate-buffered saline (PBS). Then, 15-μm-thick brain sections were acquired using a microtome. Serial coronal sections between −2.12 and −4.80 mm bregma were collected for histological analysis. Every eighth section beginning at a random start point was selected for further staining by an investigator blinded to the treatment of the animals.

### 2-BFI Administration and Experimental Groups

**FIGURE 1:**
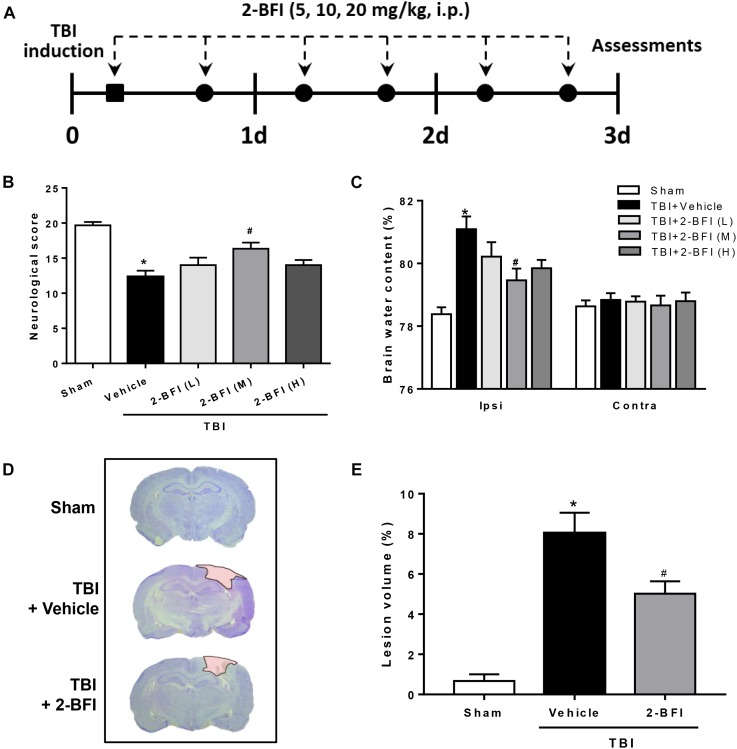
The effects of 2-BFI on neurological function, brain edema, and cortical contusion volume after TBI. **(A)** Schematic diagram of the experimental design. Rats were injured and then received twice-daily injections of 2-BFI (5, 10, 20 mg/kg) or an equal volume of vehicle for three consecutive days starting 30 min post injury. **(B)** Neurological scores were evaluated by a modified Garcia test. Treatment with 2-BFI at the median dose (10 mg/kg) improved neurological scores at 72 h after TBI. **(C)** The median dose of 2-BFI reduced TBI-induced brain edema in the ipsilateral hemisphere at 72 h post injury. **(D)** Representative photographs of cresyl violet-stained brain sections from sham, vehicle-treated, and 2-BFI (10 mg/kg)-treated TBI rats at 72 h post injury. The pink-shaded region indicates the lesioned area. **(E)** Quantitative analysis shows that 2-BFI treatment markedly decreased the contusion volume induced by TBI. *n* = 6 animals per group. Data are expressed as the mean ± SEM; ^∗^*P* < 0.05 vs. sham; ^#^*P* < 0.05 vs. TBI + vehicle.

2-BFI (Sigma-Aldrich, United States) was prepared in normal saline and administered by intraperitoneal (IP) injection at doses of 5, 10, and 20 mg/kg (Zhu et al., 2015). The total injection volume of each injection for each animal was 1 mL. Animals received a 2-BFI injection at 30 min following brain injury and then twice daily for 3 days, with injections separated by at least 6 h. Vehicle-treated animals received an identical volume of normal saline by IP injection at homologous time points as the 2-BFI injections (Figure 1A). Drug administration was performed by an investigator who was blinded to the experiment. Animals were initially divided into five study groups: sham, TBI + vehicle, TBI + 2-BFI (5 mg/kg), TBI + 2-BFI (10 mg/kg), and TBI + 2-BFI (20 mg/kg). Based on neurological tests and brain water content, 10 mg/kg 2-BFI was chosen to be given to TBI animals in the following experiments.

### Assessment of Neurological Deficits

Neurological function was assessed with the previously described modified Garcia scoring system at 72 h after TBI induction by an investigator who was blinded to the experimental design (Ni et al., 2018). The assessment consisted of seven tests covering spontaneous activity, axial sensation, vibrissae proprioception, lateral turning, symmetry of limb movement, forelimb walking, climbing, and grabbing. Each subtest was given a score ranging from 0 to 3, with a maximum score of 21 (no neurological deficits).

### Assessment of Cerebral Edema

Brain edema was estimated by measuring brain water content using the wet/dry method. At 72 h after TBI, the brains were quickly removed from the skull and separated into the ipsilateral cerebral hemisphere and the contralateral cerebral hemisphere. Each part was immediately weighed to obtain the wet weight and then placed in an oven set at 105°C for 72 h to obtain the dry weight. Brain water content was calculated using the following equation: brain water content = (wet weight – dry weight)/wet weight × 100%.

### Contusion Volume Measurement

To quantify cortical tissue loss following TBI, coronal sections spanning the rostral-caudal extent of the injured cortex were stained with cresyl violet and imaged using a digital camera integrated with a light microscope (Leica Microsystems, Germany). The area of cortical tissue loss for each section was outlined based on the Cavalieri method of stereology (Coggeshall, 1992) and quantified using NIH ImageJ software. Every eighth section was analyzed beginning at a random start point. Lesion volume was obtained by multiplying the sum of the lesion areas by the distance between sections. The percent lesion volume was calculated by dividing each lesion volume by the total ipsilateral hemisphere volume.

### Evans Blue Dye Extravasation

Blood-brain barrier permeability was determined by measuring Evans blue (EB) extravasation. At 72 h after TBI, 2.5% EB (5 mL/kg; Sigma-Aldrich, United States) was injected into the femoral vein and allowed to circulate for 1 h. Then, the rats were transcardially perfused with code saline to remove intravascular EB. The right hemisphere of the brains was collected and weighed rapidly, homogenized in 50% trichloroacetic acid, and centrifuged at 15,000 g for 30 min. The supernatant was measured for absorbance at 620 nm using a spectrophotometer. The amount of EB dye was quantified using a standard curve and expressed as μg/g of brain tissue.

### Western Blotting

Western blotting was performed as previously described (Rui et al., 2018). Briefly, proteins were extracted from the peri-injury cortex tissue by homogenization in RIPA buffer (Beyotime, China). Thirty micrograms of sample proteins were loaded and separated by electrophoresis on an 8–12% polyacrylamide gel (Bio-Rad, United States). Proteins were then transferred to PVDF membranes (Millipore, United States). The membranes were blocked with 5% non-fat milk and then probed with primary antibodies against zonula occludens (ZO)-1 (1:1000, Abcam, United States), occludin (1:1000, Invitrogen, United States), claudin-5 (1:1000, Invitrogen, United States), NLRP3 (1:1000, Abcam, United States), caspase-1 (1:500, NOVUS, United States), apoptosis-associated speck-like protein containing a caspase recruitment domain (ASC) (1:500, NOVUS, United States), IL-1β (1:500, Abcam, United States), RIP1 (1:1000, Abcam, United States), RIP3 (1:1000, Abcam, United States), MLKL (1:1000, Affinity, United States), and glyceraldehyde-3-phosphate dehydrogenase (GAPDH) (1:10000, Abcam, United States). Following TBS-T washes, the membranes were incubated in appropriate horseradish peroxidase (HRP)-conjugated secondary antibodies (1:10000, Santa Cruz, United States). The bands were visualized with enhanced chemiluminescence (Beyotime, China), and the band density was quantified using ImageJ and normalized to the expression of GAPDH as the internal loading control. The results are expressed as a relative density ratio, which was normalized to the mean value of the sham group.

### Immunofluorescence Staining

Sections were washed three times in PBS with 0.3% Triton X-100 (PBST) for 5 min each wash and blocked with 10% normal bovine serum (Gibco, United States) in PBST for 1 h at room temperature. The following primary antibodies were incubated at 4°C overnight in 5% normal goat serum (NGS) in PBST: claudin-5 (1:100, Invitrogen, United States), von Willebrand factor (vWF; 1:100, Abcam, United States), ionized calcium binding adapter molecule 1 (Iba1; 1:1000, Wako, Japan; 1:200, Abcam, United States), myeloperoxidase (MPO; 1:100, Abcam, United States), NLRP3 (1:100, Abcam, United States), RIP kinase 1 (RIPK1) (1:200, Abcam, United States), RIPK3 (1:200, Abcam, United States), and neuronal nuclei (NeuN; 1:200, Millipore, United States). Following three washes in PBST, sections were incubated with species-specific secondary antibodies conjugated to Alexa-Fluor (1:800, Invitrogen, United States) for 1 h at room temperature. Finally, nuclei were counterstained with 4’,6-diamidino-2-phenyl-indole (DAPI, Abcam, United States). The omission of the primary antibody served as a negative control. Immunofluorescence images were captured using a laser confocal microscope (Leica DMi8, Germany). Five non-overlapping regions from the perilesional area per section and three sections per animal were used for imaging. The immunofluorescence intensities of claudin-5 and vWF and the number of positive cells were determined using ImageJ software (NIH). Microglial activation was scored by evaluating the Iba1 expression intensity and microglial morphology modified from Kauppinen et al. (2008), as detailed in Table 1. All analyses were performed in a blinded manner.

**Table 1:** Scoring of microglial activation.

Cell morphology (% amoeboid shape)	Score	Cell number (cells per 100 μm^2^)	Score
None	0	None	0
1–30%	1	1–10	1
31–90%	2	11–20	2
>90%	3	>20	3

*Microglia cells were identified by Iba1 immunoreactivity. Scoring with the two criteria were combined to yield an aggregate microglia activation score (0–6).*

### Real-Time PCR

Total RNA was extracted using Trizol reagent (Invitrogen, United States). Reverse transcription (RT) was performed on 1 μg of total RNA using an RT reagent kit (Thermo Fisher, United States) according to the manufacturer’s instructions. Real-time PCR was performed using the PowerUp^TM^ SYBR^TM^ Green Master Mix kit (Thermo Fisher, United States). The primers used were as follows: IL-1β forward, GGGATGATGACGACCTGCTA; IL-1β reverse, TGTCGTTGCTTGTCTCTCCT. RIP1 forward, TCAGGACCACGGTGCCAAAGA; RIP1 reverse, ATCTCCATAGTGCTGAGCCCAACC. RIP3 forward, ACCCTGACTGTGACCCTCCCT; RIP3 reverse, TCAAGCCCTCCAATGTTCTGC. GAPDH forward, TGGCCTTCCGTGTTCCTACC; GAPDH reverse, CGCCTGCTTCACCACCTTCT; All samples were performed in triplicate. Thermal cycling conditions were set according to the manufacturer’s recommendations. The relative quantification of target mRNAs was normalized to GAPDH expression.

### Enzyme-Linked Immunosorbent Assay (ELISA)

IL-1β levels in brain tissue homogenate prepared from the pericontusional cortex were detected using the IL-1β ELISA kit (Boster Biosciences, China) in accordance with the manufacturer’s protocol.

### Cell Death Assays

Hematoxylin and eosin (H&E) staining and propidium iodide (PI) staining were used to detect cell death. After air-drying, frozen brain sections were stained with H&E according to the standard protocol. The sections were then detected under a Leica light microscope. For PI staining, a dose of 20 mg/kg PI (Sigma-Aldrich, United States) was injected into the rat femoral vein 1 h before sacrifice. Frozen brain sections were obtained as above, covered with DAPI for 20 min and then photographed under a laser confocal microscope. For quantification of necrotic cells and PI-positive cells, five different fields from the perilesional area per section and three sections per animal were analyzed using ImageJ by an investigator who was blinded to the experimental groups. The results are presented as percentages of positive cells.

### Statistical Analysis

In this study, all animals were randomly assigned to different groups, and the “*n*” denotes the number of animals in each group. All data are presented as the mean ± SEM. Statistical differences among groups were analyzed using one-way analysis of variance (ANOVA) followed by Tukey’s *post hoc* test. A *P* value of < 0.05 was considered to be statistically significant. All statistical analyses were performed using GraphPad Prism 6 (GraphPad software, United States).

## Results

### 2-BFI Administration Ameliorated Neurological Deficits, Brain Edema, and Cortical Contusion Volume After TBI

To determine whether postinjury administration of 2-BFI is protective in TBI rats, neurological deficits and brain water content were evaluated at 72 h following TBI. As evaluated by the modified Garcia tests, TBI rats who received vehicle exhibited significantly worse neurobehavioral scores than the sham group. However, treatment with the median dose of 2-BFI (10 mg/kg) significantly improved the neurological function scores of rats 72 h after TBI (*F*_(4,25)_ = 11.65, *P* < 0.05, Figure 1B). Consistently, brain water content in the left hemisphere was significantly increased by TBI in the vehicle-treated animals, but this increase was significantly reduced with the median dose of 2-BFI (*F*_(4,25)_ = 7.78, *P* < 0.05, Figure 1C). There was no significant difference in brain water content in the right hemisphere among the groups. Therefore, the median dosage of 2-BFI was used for subsequent experiments.

In addition, cresyl violet-stained brain sections from sham, vehicle-treated, and 2-BFI-treated injured rats were detected to examine the role of 2-BFI in cortical tissue loss post TBI. As shown in Figure 1D,E, sham-injured rats showed no gross lesion or noticeable damage to the cerebral cortex. In contrast, TBI rats who received vehicle exhibited a remarkably large lesion, which was significantly smaller in 2-BFI-treated animals (*F*_(2,15)_ = 26.71, *P* < 0.05 vs. TBI + vehicle).

### 2-BFI Administration Mitigated the BBB Disruption After TBI

**FIGURE 2:**
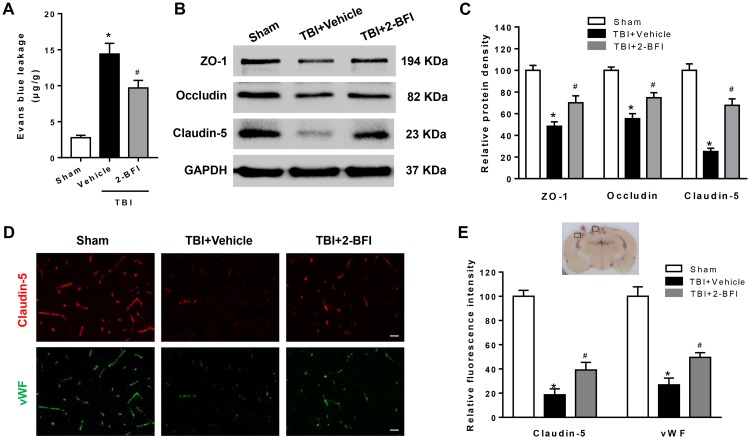
The effects of 2-BFI on BBB disruption after TBI. **(A)** Treatment with 2-BFI attenuated TBI-induced EB dye extravasation at 72 h post injury. **(B)** Representative Western bands showing levels of ZO-1, occludin, and claudin-5 in the pericontusional cortex 72 h post injury. **(C)** Quantitative analysis of Western blots shows that 2-BFI treatment reversed ZO-1, occludin, and claudin-5 degradation after TBI. **(D)** Representative photographs of immunofluorescence staining of claudin-5 (red) and vWF (green) expression in the pericontusional cortex at 72 h post injury. The right panel indicates the location of the immunofluorescence images (small black box). Bar = 25 μm. **(E)** Quantification of the fluorescence intensities showed that 2-BFI mitigated the loss of both vWF and claudin-5 immunoreactivity after TBI. *n* = 6 animals per group. Data are expressed as the mean ± SEM; ^∗^*P* < 0.05 vs. sham; ^#^*P* < 0.05 vs. TBI + vehicle.

To explore the impact of 2-BFI on BBB permeability after TBI, we examined EB dye extravasation, an indicator of BBB disruption, and the expression of tight junction proteins. There was remarkable EB extravasation in the ipsilateral hemisphere at 72 h following TBI, and 2-BFI-treated rats exhibited significantly less EB leakage in the ipsilateral hemisphere than vehicle-treated TBI rats (*F*_(2,15)_ = 75.26, *P* < 0.05, Figure 2A). Consistent with the EB content test, Western blotting analysis of the peri-injury cortex showed that the expression of tight junction proteins ZO-1, occludin, and claudin-5 were markedly lower in the vehicle-treated group than in the sham group, and this reduction was rescued with 2-BFI treatment (*F*_(2,15)_ = 25.21 for ZO-1, *F*_(2,15)_ = 28.28 for occludin, *F*_(2,15)_ = 51.49 for claudin-5, *P* < 0.05, Figure 2B,C). In addition, immunofluorescence staining of vWF (a marker of endothelial cells) and claudin-5 showed that TBI caused the loss of both vWF and claudin-5 immunoreactivity in brain tissue from the perilesional area, and this loss was also significantly mitigated after 2-BFI treatment (*F*_(2,15)_ = 38.51 for vWF, *F*_(2,15)_ = 60.67 for claudin-5, *P* < 0.05, Figure 2D,E).

### 2-BFI Administration Inhibited Microglia Activation, Neutrophil Infiltration, and Inflammatory Cytokine Secretion After TBI

**FIGURE 3:**
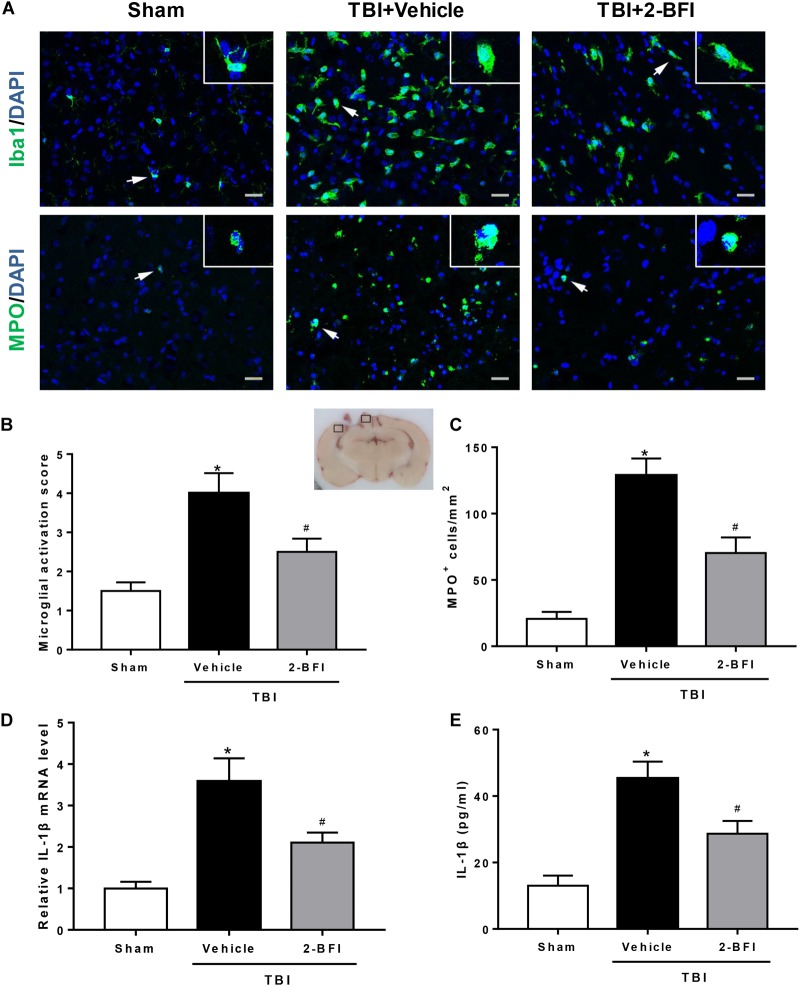
The effects of 2-BFI on microglia activation, neutrophil infiltration, and IL-1β secretion after TBI. **(A)** Representative immunofluorescence staining images of Iba1 (green) and MPO (green) in the pericontusional cortex at 72 h after TBI. Bar = 25 μm. **(B)** Quantification of microglial activation based on the scores for both microglial number and morphology. **(C)** Quantitative analysis shows that 2-BFI inhibited the increase in the number of MPO-positive cells after TBI. **(D)** 2-BFI inhibited the elevation in IL-1β mRNA levels in the pericontusional cortex at 72 h after TBI. **(E)** TBI-induced IL-1β secretion was also reduced by 2-BFI treatment. *n* = 6 animals per group. Data are expressed as the mean ± SEM; ^∗^*P* < 0.05 vs. sham; ^#^*P* < 0.05 vs. TBI + vehicle.

Next, we investigated the impact of 2-BFI on neuroinflammation after TBI. Immunofluorescence staining was performed to determine Iba1 (a marker of microglia) and MPO (a marker of neutrophil infiltration) expression in brain tissue. As shown in Figure 3A, after brain trauma, microglia appear activated: increased cell size, ameboid shape, and intensified Iba1 staining. Treatment with 2-BFI significantly reduced microglial activation score relative to vehicle treatment (*F*_(2,15)_ = 10.96, *P* < 0.05, Figure 3A,B). In addition, compared to the sham group, vehicle-treated TBI animals showed a significant increase in the number of MPO-positive cells in the peri-injury cortex, which was significantly inhibited by 2-BFI treatment at 72 h after TBI (*F*_(2,15)_ = 26.78, *P* < 0.05, Figure 3A,C). Furthermore, real-time PCR and ELISA analyses showed that IL-1β levels in the peri-injury cortex were markedly reduced in the 2-BFI-treated animals compared to those in the vehicle-treated group at 72 h after TBI (*F*_(2,15)_ = 27.99 for PCR, *F*_(2,15)_ = 39.57 for ELISA, *P* < 0.05, Figure 3D,E).

### 2-BFI Administration Inhibited NLRP3 Inflammasome Activation and Subsequent IL-1β Production After TBI

**FIGURE 4:**
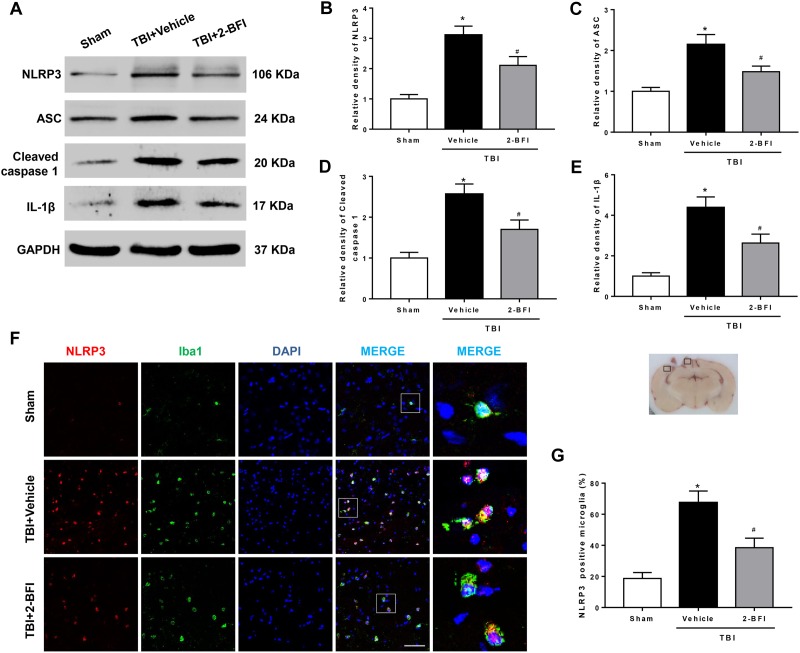
The effects of 2-BFI on NLRP3 inflammasome activation and subsequent IL-1β production after TBI. **(A)** Representative Western bands showing the levels of NLRP3, ASC, caspase-1, and IL-1β in the pericontusional cortex at 72 h after TBI. **(B–E)** Quantification of Western blots shows that 2-BFI inhibited the TBI-induced upregulation of NLRP3 inflammasome component expression and IL-1β production. **(F)** Representative images of double immunofluorescence staining for NLRP3 (green) with Iba1 (red)-labeled microglia in the pericontusional cortex at 72 h after TBI. Nuclei were counterstained with DAPI (blue). Bar = 50 μm. **(G)** Quantification of NLRP3-positive microglia shows that 2-BFI reduced TBI-associated NLRP3 overexpression in microglia. *n* = 6 animals per group. Data are expressed as the mean ± SEM; ^∗^*P* < 0.05 vs. sham; ^#^*P* < 0.05 vs. TBI + vehicle.

We further examined whether the NLRP3 inflammasome was involved in the protective effect of 2-BFI after TBI. Western blot results revealed that the protein levels of NLRP3 inflammasome components, including NLRP3, ASC and caspase-1, and the levels of IL-1β release were evidently elevated in the vehicle group compared to those in the sham group at 72 h post TBI. TBI rats treated with 2-BFI exhibited a significant reduction in NLRP3 inflammasome component expression and a subsequent secretion of mature IL-1β compared to vehicle-treated animals (*F*_(2,15)_ = 17.86 for NLRP3, *F*_(2,15)_ = 11.12 for ASC, *F*_(2,15)_ = 13.88 for caspase-1, *F*_(2,15)_ = 17.31 for IL-1β, *P* < 0.05, Figure 4A–E). Furthermore, immunofluorescence analysis confirmed that the increased NLRP3 immunoreactivity in microglia from the perilesional cortex post injury was significantly attenuated in 2-BFI-treated animals (*F*_(2,15)_ = 16.67, *P* < 0.05, Figure 4F,G).

### 2-BFI Administration Reduced Necrotic Cell Death in the Pericontusional Cortex After TBI

**FIGURE 5:**
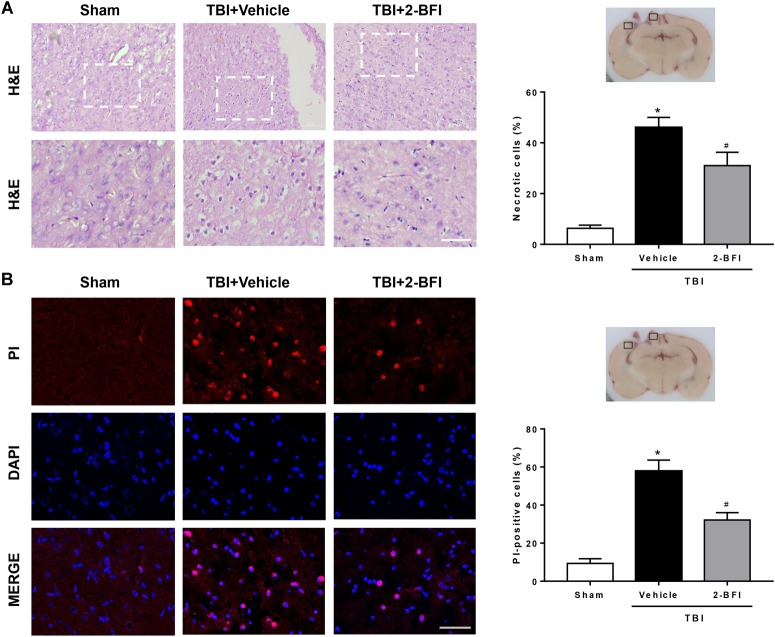
The effects of 2-BFI on necrotic cell death in the pericontusional cortex after TBI. **(A)** Representative H&E-stained images of the perilesional region in brain sections from sham, vehicle-treated, and 2-BFI-treated TBI rats at 72 h post injury. Areas outlined in white are enlarged in the lower panels. Bar = 50 μm. Quantitative analysis shows that treatment with 2-BFI markedly reduced the number of necrotic cells compared with vehicle treatment after TBI. **(B)** Representative fluorescence micrographs of PI labeling in the experimental groups. Nuclei were counterstained with DAPI (blue). Bar = 50 μm. 2-BFI treatment reduced the number of PI-positive cells in the pericontusional cortex compared with vehicle treatment at 72 h after TBI. *n* = 6 animals per group. Data are expressed as the mean ± SEM; ^∗^*P* < 0.05 vs. sham; ^#^*P* < 0.05 vs. TBI + vehicle.

To estimate the effect of 2-BFI treatment on brain damage at 72 h following TBI induction, we assessed neural cell death in perilesional brain tissue and performed H&E staining to examine neuronal morphology. As shown in Figure 5A, normal neurons exhibited round and pale nuclei, whereas neurons undergoing necrotic cell death showed pyknotic or vacuolated nuclei with intact extracellular membranes. In the sham-operated group, very few necrotic cells were observed in the brain. Conversely, many necrotic cells were observed in the peri-injury cortex at 72 h following TBI, but the number of necrotic cells was remarkably reduced after 2-BFI treatment (*F*_(2,15)_ = 26.33, *P* < 0.05). In addition, necrotic cell death was also detected by *in vivo* PI labeling. The results showed that TBI triggered a significant increase in the number of PI-positive cells in the vehicle group, which was markedly attenuated by 2-BFI treatment (*F*_(2,15)_ = 32.05, *P* < 0.05, Figure 5B).

### 2-BFI Treatment Inhibited RIP1, RIP3, and MLKL Expression in the Pericontusional Cortex After TBI

**FIGURE 6:**
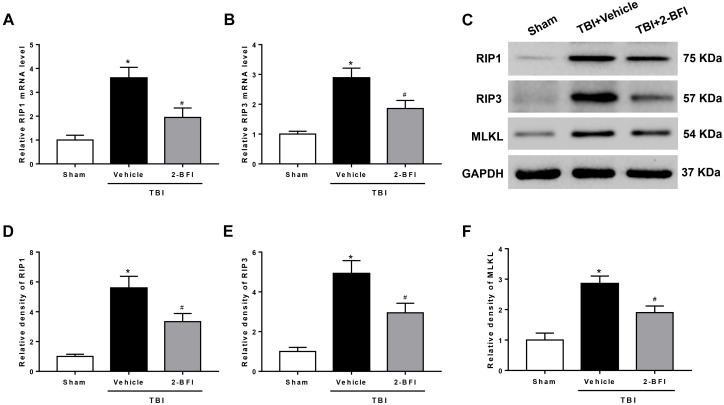
The effects of 2-BFI on RIP1, RIP3, and MLKL expression after TBI. Quantitative real-time PCR analysis shows that TBI-induced RIP1 **(A)** and RIP3 **(B)** mRNA overexpression in the pericontusional cortex was inhibited by 2-BFI treatment. **(C)** Representative Western bands showing the protein expression of RIP1, RIP3, and their substrate MLKL in the pericontusional cortex at 72 h after TBI. **(D–F)** Quantitative analysis of Western blots shows that 2-BFI blocked the elevation in the expression of these necroptosis-associated proteins after TBI. *n* = 6 animals per group. Data are expressed as the mean ± SEM; ^∗^*P* < 0.05 vs. sham; ^#^*P* < 0.05 vs. TBI + vehicle.

To further demonstrate the inhibitory effect of 2-BFI on necroptosis, we detected the expression of necroptosis-related proteins in the peri-injury cortex. As evaluated by real-time PCR, upregulation of RIP1 and RIP3 mRNA levels was observed 72 h after TBI in the vehicle group when compared to that in the sham group. 2-BFI treatment significantly reduced the levels of both mRNAs relative to vehicle treatment (*F*_(2,15)_ = 32.28 for RIP1, *F*_(2,15)_ = 13.93 for RIP3, *P* < 0.05, Figure 6A,B). Consistently, Western blot results revealed that the protein levels of RIP1, RIP3 and their substrate MLKL were all significantly elevated in TBI rats that received vehicle treatment and that 2-BFI treatment markedly decreased the expression of these proteins compared to vehicle treatment (*F*_(2,15)_ = 16.48 for RIP1, *F*_(2,15)_ = 17.31 for RIP3, *F*_(2,15)_ = 15.67 for MLKL, *P* < 0.05, Figure 6C–F).

### 2-BFI Attenuated RIP1 and RIP3 Expression in Neurons of the Pericontusional Cortex After TBI

**FIGURE 7:**
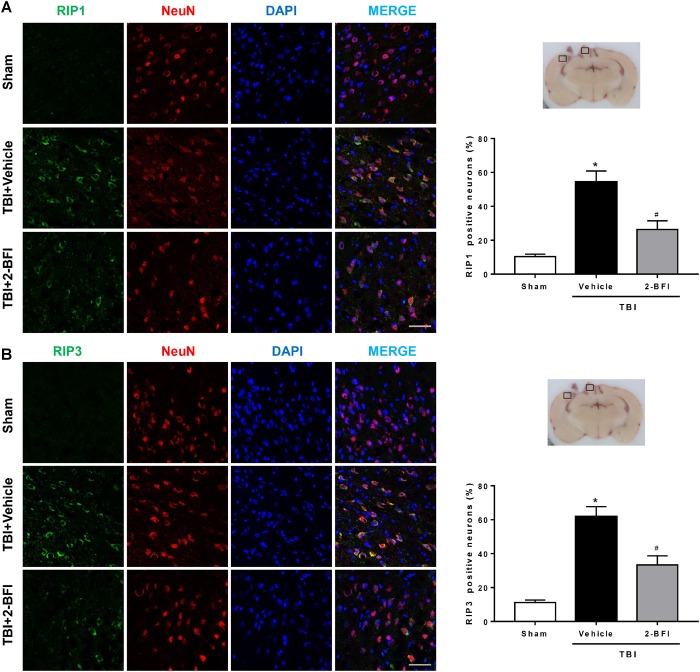
The effects of 2-BFI on RIP1 and RIP3 expression in neurons of the pericontusional cortex after TBI. Representative images of immunofluorescent staining of RIP1 **(A)** and RIP3 **(B)** (green) with NeuN (red)-marked neurons in the pericontusional cortex at 72 h after TBI. Nuclei are stained with DAPI (blue). Bar = 50 μm. Quantification of RIP1- and RIP3-positive neurons shows that 2-BFI treatment reduced the expression of RIP1 and RIP3 in neurons in the pericontusional cortex 72 h after TBI. *n* = 6 animals per group. Data are expressed as the mean ± SEM; ^∗^*P* < 0.05 vs. sham; ^#^*P* < 0.05 vs. TBI + vehicle.

We then detected the effects of 2-BFI on the cellular localization of RIP1 and RIP3, two critical mediators of necroptosis, by immunofluorescence staining with NeuN. As shown in Figure 7, the immunoreactivities of RIP1 (Figure 7A) and RIP3 (Figure 7B) were mainly located in the cell membranes and generally colocalized within neurons in brain tissue. In the sham group, few RIP1- and RIP3-positive neurons were detected in the brain. In contrast, significantly more RIP1- and RIP3-positive neurons were observed in the pericontusional cortex in the vehicle group 72 h post TBI, but this increase was markedly inhibited by 2-BFI treatment (*F*_(2,15)_ = 31.18 for RIP1, *F*_(2,15)_ = 36.06 for RIP3, *P* < 0.05).

## Discussion

TBI is now widely acknowledged to be a highly complex disease process that is characterized by multiple cellular and molecular pathological events (Nizamutdinov and Shapiro, 2017). Thus, there is a general consensus that drugs that selectively target a single injury factor may not be as effective as multitarget pharmacological treatment in outcome improvements after TBI (Kumar and Loane, 2012; Loane et al., 2015). In this sense, 2-BFI might serve as a potential drug for the treatment of TBI due to its protective effects against both inflammation and programmed cell death, two important mechanisms in the progression of TBI (Li, 2017; Tian et al., 2017b; Siemian et al., 2018). The present study revealed three major findings: (1) postinjury administration of 2-BFI attenuated neurological deficits, brain edema, BBB permeability and cortical tissue loss in a rat model of TBI; (2) 2-BFI treatment reduced microglial activation, neutrophil infiltration, and proinflammatory cytokine IL-1β secretion, which is related to NLRP3 inflammasome activation after TBI; (3) the TBI-induced increase in necroptosis and necroptosis-associated proteins, including RIP1, RIP3, and MLKL expression, was repressed by 2-BFI treatment.

The degree of posttraumatic brain edema is one of the main determinants of survival and neurological outcome after TBI, and a key manifestation of edema is the disruption of the BBB (Donkin and Vink, 2010; Winkler et al., 2016). Thus, alleviating posttraumatic edema by protecting the BBB has become a focus of recent research to improve neurofunction after TBI (Shlosberg et al., 2010). The main structures responsible for the properties of the BBB are cerebrovascular endothelial cells, which are connected to an integrated vascular system by continuous intercellular tight junctions. The loss of the expression of tight junction proteins following trauma contributes to BBB breakdown and the ensuing brain edema (Shlosberg et al., 2010; Chodobski et al., 2011). In the current study, we found that treatment with 2-BFI attenuated the TBI-induced neurodeficits, brain edema and elevated BBB permeability at 72 h post injury, indicating potential vascular protection during the acute phase of TBI. Further investigation showed that administration of 2-BFI attenuated the disruption of the BBB by preserving the expression of tight junction proteins, especially claudin-5, in endothelial cells after TBI, which is consistent with the observations in a cerebral ischemia model.

Excessive posttraumatic neuroinflammation has been characterized as a major contributor to the pathology of BBB disruption and brain edema (Chodobski et al., 2011; Kumar and Loane, 2012). In particular, as an immediate consequence of the trauma, a large number of peripheral neutrophils infiltrate the brain tissue through the damaged BBB, subsequently facilitating a disproportionate activation of resident immune cells such as microglia, leading to continuous tissue injury and pervasive disruption of BBB integrity (Glushakova et al., 2014; Hellewell et al., 2016). Consistent with previous studies, we found evidence of a robust neuroinflammatory response after TBI, as indicated by an increased number of neutrophils as well as microglial activation at 72 h post injury. However, treatment with 2-BFI post trauma significantly attenuated many of these effects, supporting a critical role for 2-BFI in decreasing neuroinflammation. Indeed, 2-BFI has been shown to exert antiinflammatory activity via reducing the expression of inflammatory factors (Zhu et al., 2015; Tian et al., 2017b; Siemian et al., 2018). As a key proinflammatory cytokine, IL-1β has long been speculated to be involved in posttraumatic neuroinflammation. Human and rodent studies have shown upregulation of IL-1β within hours post TBI in both brain parenchyma and cerebrospinal fluid, and this upregulation is associated with poor clinical outcomes (Ciallella et al., 2002; Winter et al., 2002). During the inflammatory process, IL-1β has the ability to amplify TBI-induced inflammatory damage by inducing further production of proinflammatory cytokines, microglial activation, and leukocyte recruitment, culminating in the expansion of pericontusional edema and thereby exacerbating brain damage (Ziebell and Morganti-Kossmann, 2010). In our study, the persistent repression of IL-1β production paralleled the reduction in the number of neutrophils and the score of microglial activation in 2-BFI-treated TBI animals, a noteworthy observation that may underline the central role of IL-1β in posttraumatic inflammation and 2-BFI neuroprotection. Recently, the NLRP3 inflammasome, a core component of the innate immune system, has been highlighted to mediate the maturation and secretion of proinflammatory cytokines, especially IL-1β, and therefore play a critical role in the initiation and amplification of the inflammatory response (Walsh et al., 2014). This inflammasome, composed of NLRP3, ASC and caspase-1, is a multiprotein complex that mediates the cleavage of caspase-1, which in turn facilitates the maturation of IL-1β. In fact, several rodent studies have reported that blockade or inhibition of NLRP3 inflammasome activation post trauma alleviates neuroinflammation and improves the histopathological and functional outcomes of TBI (Irrera et al., 2017; Ismael et al., 2018; Xu et al., 2018). In agreement, we found that 2-BFI treatment significantly suppressed TBI-induced activation of the NLRP3 inflammasome, in line with its improvement of brain edema and neurological deficits. More importantly, our histological evaluations confirmed that microglia were the main target of the inhibitory action of 2-BFI on NLRP3 inflammasome expression, further demonstrating the effectiveness of 2-BFI in reducing microglia-mediated neuroinflammation after TBI.

In addition, we used cresyl violet staining to show that TBI-damaged rats exhibited dramatic cortical tissue loss, but 2-BFI administration partially reversed this alteration in rats. The formation of tissue damage is related to the rapid necrotic cell death found in the affected regions after TBI (Raghupathi, 2004; Werner and Engelhard, 2007). For many years, necrosis has been described as a passive and accidental process caused by pathological damage. However, accumulating evidence now suggests that necrotic cell death may also be accomplished by a set of signal transduction pathways and execution mechanisms, which are viewed as appealing targets for therapeutic intervention (Vandenabeele et al., 2010). In our study, stereology analyses showed that 2-BFI significantly reduced posttraumatic neuronal loss and the number of PI-positive cells in the peri-injury cortex, indicating that 2-BFI suppressed necrotic cell death in the early stage after TBI. To further explore the possible mechanism of this phenomenon, we focused on necroptosis and associated necroptotic proteins. Necroptosis is the best-described form of programmed necrosis at present and is widely recognized as a component of caspase-independent cell death mediated by RIP1 and RIP3. Typically, RIP1 interacts with RIP3 to form a necrosis signaling complex, also called necrosome, resulting in the recruitment and activation of its downstream effectors, such as MLKL, with subsequent necroptotic cell death (Li et al., 2012a; Sun et al., 2012). In fact, the protein expression levels of RIP1 and RIP3 are elevated in the hours to days following the TBI procedure, together with an increase in contusion necrosis (Liu et al., 2018), thus suggesting that the activation of RIP1/RIP3-mediated necroptotic signaling is part of a common acute event that leads to the histopathological appearance of cell death after TBI. In sight of this, different observations have indicated that targeting the associated necroptotic proteins is an attractive approach for developing TBI therapy. For example, treatment with necrostatin-1 (Nec-1), a specific inhibitor of RIP1, has been demonstrated to reduce the amount of injured cells and tissue damage after TBI in rodents (You et al., 2008). Moreover, PIP3 knockout mice exhibit reduced posttraumatic neuronal loss and even improved functional outcomes relative to an isolated TBI (Liu et al., 2018). Similar to these findings, the TBI brains in our study showed increased protein expression of RIP1, RIP3, and their substrate MLKL, which was strongly prevented by 2-BFI treatment. Furthermore, in this study, we demonstrated that 2-BFI treatment notably reduced the expression of core molecules of necroptosis in neurons in the perilesional area. Collectively, our findings provide a new anti-programmed cell death mechanism for 2-BFI, which specifically suppresses neuronal necroptosis mediated by RIP1/RIP3 signaling and thereby reduces neuronal loss following TBI.

Interestingly, emerging evidence indicates that necroptosis can also trigger an inflammatory response by releasing intracellular components, called damage-associated molecular patterns, into the extracellular space via the ruptured plasma membrane (Chan et al., 2015; Wallach et al., 2016; Yang et al., 2018). In addition to suppressing neuronal necrosis, pharmacological inhibition of RIP1 has been shown to reduce brain neutrophil influx and microglial activation in a controlled cortical impact mouse model of TBI (You et al., 2008). Moreover, RIP3 knockout prevented the IL-1β generation associated with NLRP3 inflammasome activation and alleviated TBI-induced inflammatory injury in mice (Liu et al., 2018). These findings indicated an active role of RIP1/RIP3-mediated necroptosis in the regulation of inflammation after TBI. Thus, we cannot exclude the possibility that the antinecroptotic properties of 2-BFI in our study may also contribute to its observed inhibitory action on inflammation, and further investigation is necessary.

There are some limitations in the present study. First, we only evaluated the neuroprotective functions of 2-BFI during an acute stage post TBI, while the long-term outcomes were not investigated in the present study. Second, the experiment was not designed to study other actions of 2-BFI, such as antioxidant activity (Tian et al., 2017a,b). Additionally, the effects of 2-BFI on other inflammatory cytokines, such as tumor necrosis factor (TNF)-α and IL-17 (Zhu et al., 2015; Siemian et al., 2018), were not evaluated in this study. Therefore, the above issues need to be clarified in future studies.

In summary, the findings of this study add new evidence to the neuroprotective effects of 2-BFI. Following TBI, 2-BFI inhibits NLRP3 inflammasome-induced inflammation, which may play an important role in BBB disruption and brain edema; meanwhile, 2-BFI may reduce the posttraumatic cortical tissue loss associated with RIP1/RIP3-mediated necroptosis in the acute phase of TBI. In light of the increasing evidence of neuroprotection induced by 2-BFI, 2-BFI may have important clinical potential for the management of TBI.

## Data Availability

The raw data supporting the conclusions of this manuscript will be made available by the authors, without undue reservation, to any qualified researcher.

## Ethics Statement

This study was carried out in accordance with the ARRIVE Guidelines (Animal Research: Reporting of *In Vivo* Experiments). All animal experiments are strictly in accordance with the guideline of Soochow University Institutional Animal Care and Use Committee. The protocol was approved by the Animal Care and Use Committee of Soochow University.

## Author Contributions

HL and GC designed the research. HN, QR, and DL carried out the entire experiments. XL and DL analyzed the data. HN and QR wrote the manuscript.

## Conflict of Interest Statement

The authors declare that the research was conducted in the absence of any commercial or financial relationships that could be construed as a potential conflict of interest.
